# Reaction classification and yield prediction using the differential reaction fingerprint DRFP

**DOI:** 10.1039/d1dd00006c

**Published:** 2022-01-21

**Authors:** Daniel Probst, Philippe Schwaller, Jean-Louis Reymond

**Affiliations:** Department of Chemistry and Biochemistry, University of Bern Freiestrasse 3 3012 Bern Switzerland daniel.probst@dcb.unibe.ch jean-louis.reymond@unibe.ch; IBM Research – Europe Säumerstrasse 4 8803 Rüschlikon Switzerland

## Abstract

Predicting the nature and outcome of reactions using computational methods is a crucial tool to accelerate chemical research. The recent application of deep learning-based learned fingerprints to reaction classification and reaction yield prediction has shown an impressive increase in performance compared to previous methods such as DFT- and structure-based fingerprints. However, learned fingerprints require large training data sets, are inherently biased, and are based on complex deep learning architectures. Here we present the differential reaction fingerprint *DRFP*. The *DRFP* algorithm takes a reaction SMILES as an input and creates a binary fingerprint based on the symmetric difference of two sets containing the circular molecular *n*-grams generated from the molecules listed left and right from the reaction arrow, respectively, without the need for distinguishing between reactants and reagents. We show that *DRFP* performs better than DFT-based fingerprints in reaction yield prediction and other structure-based fingerprints in reaction classification, reaching the performance of state-of-the-art learned fingerprints in both tasks while being data-independent.

## Abstract 2

Differential Reaction Fingerprint DRFP is a chemical reaction fingerprint enabling simple machine learning models running on standard hardware to reach DFT- and deep learning-based accuracies in reaction yield prediction and reaction classification.

## Graphical abstract

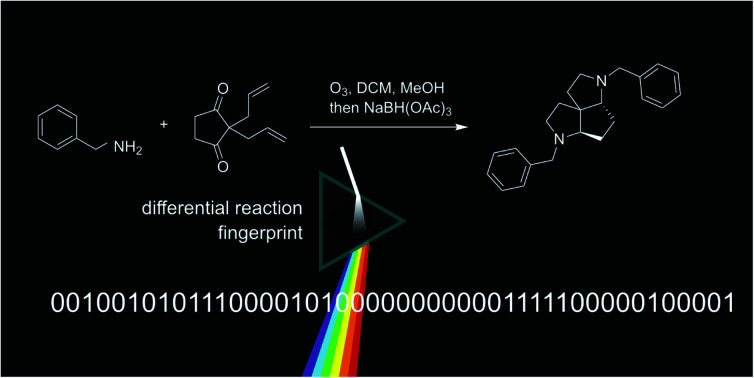

## 1 Introduction

Computational methods to predict the nature and outcome of reactions are important tools to accelerate chemical research.^1–11^ The nature of a reaction is well-described by its name and class, where a reaction class is defined by the general reaction-type and the participating chemical entities.^12–14^ Automating the classification of reactions provides a tool for chemists to search databases and to quickly evaluate and optimise a novel reaction based on the nature of similar reactions. However, commercially available reaction classifiers are based on static expert-curated transformation rules, which makes them prone to misclassifications on noisy data sets, such as reactions mined from literature.^15,16^ With the availability of ever larger reaction data sets, the use of data-driven reaction classification schemes became feasible. Data-driven reaction classification consists of two parts: the embedding of reactions as a vector, often called a fingerprint, into a metric space; and the training of machine-learning classifiers on these reaction fingerprints under the assumption they cluster according to their classes. Among the drawbacks of this generation of data-driven methods are the need to separate reactants from reagents, requiring a mapping of atoms across the two sides of a reaction equation, and a fixed number of reaction participants in order to embed the reactions in a meaningful way.^17–19^ Recently, these limitations were overcome by applying a natural language processing-inspired transformer architecture as a means to learn the vector-embedding of reactions.^11^ However, this approach comes with several drawbacks as well: training such a learned fingerprint requires large amounts of data of acceptable quality, the trained model must be retrained when new data becomes available, and the training and evaluation of the model requires specialised hard- and software to become computationally tractable—posing a challenge to accessibility and reproducibility. Due to the nature of artificial neural networks (ANNs), learned fingerprints are also challenging to interpret. In the case of the transformer-based model introduced by Schwaller *et al.*, a careful analysis of attention weights is required.^11^

An important outcome of a chemical reaction is its yield, the percentage of successfully converted reactants into the desired product. Computational methods for predicting such yields are highly valuable in synthesis-planning, where high yields are of paramount importance-especially in multi-step reactions. Earlier work used physics-based descriptors or structure-based molecular fingerprints to classify chemical reactions or predict reaction yields.^6,17,19^ While physics-based descriptors require compute-intensive calculations that involve the approximation of the N-body wave function of molecules, structure-based descriptors that are calculated from the molecular graph fail to generalize between data sets.^11^ Similar to the problem of reaction classification, the recent availability of large data sets and the resurgence of ANNs, deep learning-based learned fingerprints have been introduced as an alternative to earlier methods, outperforming them by considerable margins.^10^ However, these methods also suffer the same drawbacks as their counterparts used for reaction classification.

Here we report a molecular fingerprint for chemical reactions called differential reaction fingerprint (*DRFP*), which is computed from circular substructures in the reaction SMILES without the need for a training data set. Compared to the approach introduced by Schneider *et al.*,^17^*DRFP* does not apply weights based on atom-mapping to differentiate between reactants and reagents, does not require the calculation of molecular properties for the reagents, and does not apply arithmetic operations on individual molecular fingerprints, such as the atom pair fingerprint. We show that *DRFP* performs as well as learned fingerprints for the tasks of reaction classification and yield prediction.

## 2 Results and discussion

### 2.1 Fingerprint design

**Fig. 1:**
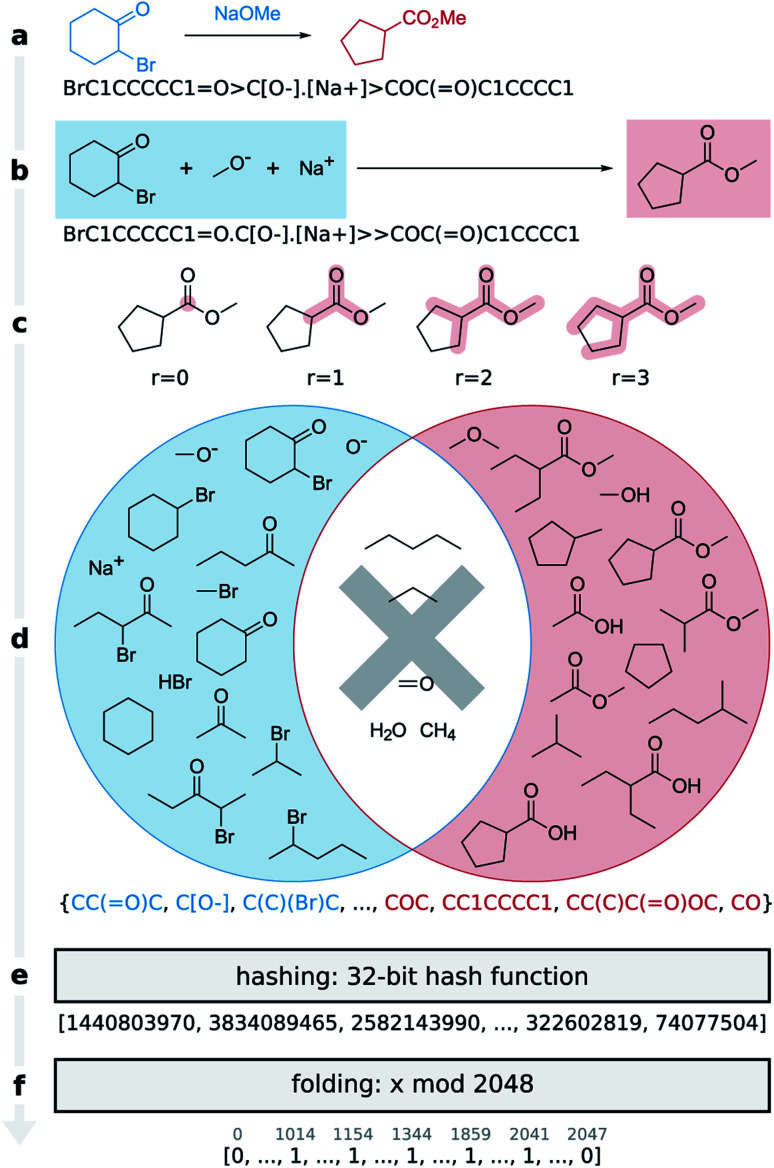
Encoding a Favorskii rearrangement as a *DRFP* fingerprint. (a) The input reaction where the reactant is written to the left, the reagent above, and the product to the right of the arrow. The respective SMILES representation, which is used as the input is written below. (b) *DRFP* does not separate reactants and reagents, a distinction which is often ambiguous. This is reflected in the reaction drawing and the associated SMILES, where the reagents have been moved to the reactants. (c) The algorithm extracts all circular substructures with radii of 0, 1, 2, and 3, as well as all rings from the reactants and products, and stores them as two SMILES-encoded sets of molecular *n*-grams (d). Next, the symmetric difference of the two sets is calculated and stored as the final set. (e) This final set of molecular *n*-grams is then hashed to a vector of 32-bit integers and (f) folded into a fixed-length binary vector using a modulo operation.

Here we present the differential reaction fingerprint (*DRFP*) for reaction search and categorization as well as yield prediction. The reaction fingerprint *DRFP* borrows the creation of circular substructures from a molecule and the subsequent hashing of their SMILES representations from the chemical fingerprints ECFP and MHFP, respectively (see Fig. 1 and molecular *n*-grams), where circular substructures for a molecule are created by extracting the neighbourhoods of a given radius *r* from the molecular graph for each atom in the molecule.^20,21^ However, as reaction SMILES consist of multiple molecules in the form REACTANTS > REAGENTS > PRODUCTS (Fig. 1a), three additional steps have to be introduced: (I) the reagents are added to the reactants, resulting in the representation REACTANTS + REAGENTS ≫ PRODUCTS (Fig. 1b); (II) circular substructures are extracted from each molecule (Fig. 1c), resulting in two sets of molecular *n*-grams *R* and *P* (Fig. 1d, red and blue circles); (III) the symmetric difference of the two sets *S* = *R*Δ*P* is taken (Fig. 1d, shaded areas of circles), hashed using an arbitrary hash function with a sufficiently low collision probability (Fig. 1e), and then folded into a fix-length binary vector using the hash function *h*(*k*) = *k* mod *d*, where *k* ∈ *S*, and *d* is the desired dimensionality of the fingerprint (Fig. 1f). Hashing and folding are required to transform the set of SMILES, which can differ in cardinality given different input reactions, into a binary vector of predefined dimensionality *d* that is independent from the input reaction. Binary vectors require little space in memory and can be processed by most machine learning methods.

Similar to the transformer-based learned fingerprint, *DRFP* does not distinguish between reactants and reagents, and accepts an arbitrary number of molecules on both sides of the chemical equation. Given this conceptually simple fingerprint, we show that its performance, when applied to tasks mentioned in the introduction, rivals or even surpasses that of state-of-the-art methods while using minimal non-specialised computational resources and no specialised hard- or software (see Computational resources). The fingerprint requires an unannotated, non-atom-mapped reaction SMILES as input and embeds this molecular representation from reaction SMILES space into an arbitrary low dimensional binary metric space through set operations and subsequent hashing and folding. We show that a *k*-NN classifier trained with *DRFP* outperforms those trained on existing, non-learned fingerprints and rivals or surpasses the performance of learned fingerprints without the need for supervised learning pre-classification. Furthermore, the fingerprint can act as an unbiased benchmark for new methods. Finally, we show that this method, based on a simple set operation and hashing scheme, can perform better than both deep learning-based learned fingerprints and physics-based descriptors in yield prediction tasks. We make the fingerprint creation algorithm available as a pypi package (drfp). The source code, data, and documentation are available on GitHub (https://github.com/reymond-group/drfp).

### 2.2 Reaction classification

As a reaction classification task, we investigated the open-source USPTO 1k TPL data set, which we previously introduced.^11^ In USPTO 1k TPL, the reaction classes were generated by extracting the 1000 most common templates from the USPTO data set.^22^ Atom-maps that are required to extract templates were predicted using RXNMapper.^23^ The task is to predict the corresponding template class given a chemical reaction.

**Fig. 2:**
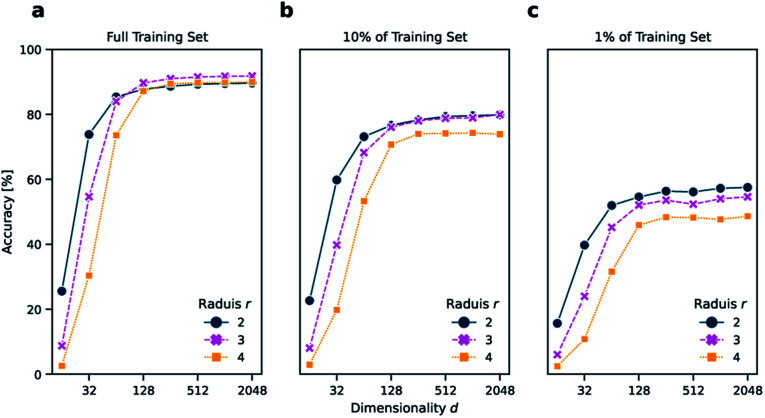
Accuracy of the *k*-nearest neighbor classification on (a) the entire TPL data set, (b) 10% of the data set, and (c) 1% of the data set using *DRFP* fingerprints for dimensionality *d* ∈ {16, 32, 64, 128, 256, 512, 1024, 2048} and *r* ∈ {2, 3, 4}. The accuracy starts to plateau with *d* = 128 independently from the amount of training data. However, a lower *r* increases the accuracy in low data settings and when a low dimensionality *d* is chosen due to increased generality and fewer collisions, respectively.

The reaction classification was carried out using the *k*-nearest neighbor classifier based on faiss^24^ as defined by Schwaller *et al.*^11^ Initially, different versions of *DRFP* were evaluated on the USPTO 1k TPL set using a number of different configurations, namely for radius *r* ∈ {2, 3, 4} and dimensionality *d* ∈ {16, 32, 64, 128, 256, 512, 1024, 2048}. For all chosen radii, the accuracy increases strongly between *d* = 16 to *d* = 128, while only increasing slightly from *d* = 256 to *d* = 2048. The *r* = 2 variant performs better than *r* ∈ {3, 4} for *d* ∈ {16, 32} (Fig. 2a). This is due to fewer collisions during mod hashing resulting from fewer extracted sub-structures. Starting with *d* = 256, the *r* = 3 variant performs better than both the other variants.

Reducing the training set to 10 and 1% of its original size, aside from a general reduction in accuracy, also leads to a better relative performance of the *r* = 2 variant across all dimensions *d* (Fig. 2b and c). These results suggest that choosing the *r* = 2 variant might be advantageous in low data settings, and there is no value in choosing *r* = 4 over *r* = 2 or *r* = 3, independent from *d* and the amount of available training data. However, as the *r* = 3 variant performed best in the case of the complete training set for high *d*, the *r* = 3 and *d* = 2048 variant is chosen for all further benchmarks, including reaction yield predictions.

Table 1 shows the classification accuracy of *DRFP* on the USPTO 1k TPL data set compared to the structure-based fingerprint AP3 256 and the learned fingerprint rxnfp.^11,17^ Evaluating the *k*-nearest neighbour classification benchmark on the TPL data set, *DRFP* outperforms the structure-based fingerprint AP3 256 by a factor of 3.1 and reaches 93% of the performance of the learned fingerprint rxnfp. In addition, a variant of *DRFP* that mimics the subtraction method of AP3 256 is evaluated, performing better than AP3 256 but not reaching the performance of the symmetric difference-based variant of *DRFP*. Replacing the *k*-nearest neighbour classifier with a simple multilayer perceptron (MLP) for *DRFP* and AP3 256, *DRFP* reaches 99% of the performance of rxnfp, while AP3 256 only reaches 82%. This result suggests that conceptual complexity, including learning, can be factored out of fingerprint creation, moving it instead to the classification task with a minor impact on classification performance. A non-learned fingerprint has the advantages of reducing bias and increasing the interpretability of results as each feature can be mapped to one or more molecular substructures.

**Table 1:** Reaction classification accuracy on the USPTO 1k TPL data set

USPTO 1k TPL	Classifier	Accuracy	CEN	MCC
rxnfp	5-NN	**0.989**	0.006	0.989
AP3 256	5-NN	0.295	0.242	0.292
*DRFP (subtraction)*	5-NN	0.851	0.850	0.074
*DRFP*	5-NN	0.917	0.041	0.917
AP3 256	MLP	0.809	0.101	0.808
*DRFP*	MLP	0.977	0.011	0.977

**Fig. 3:**
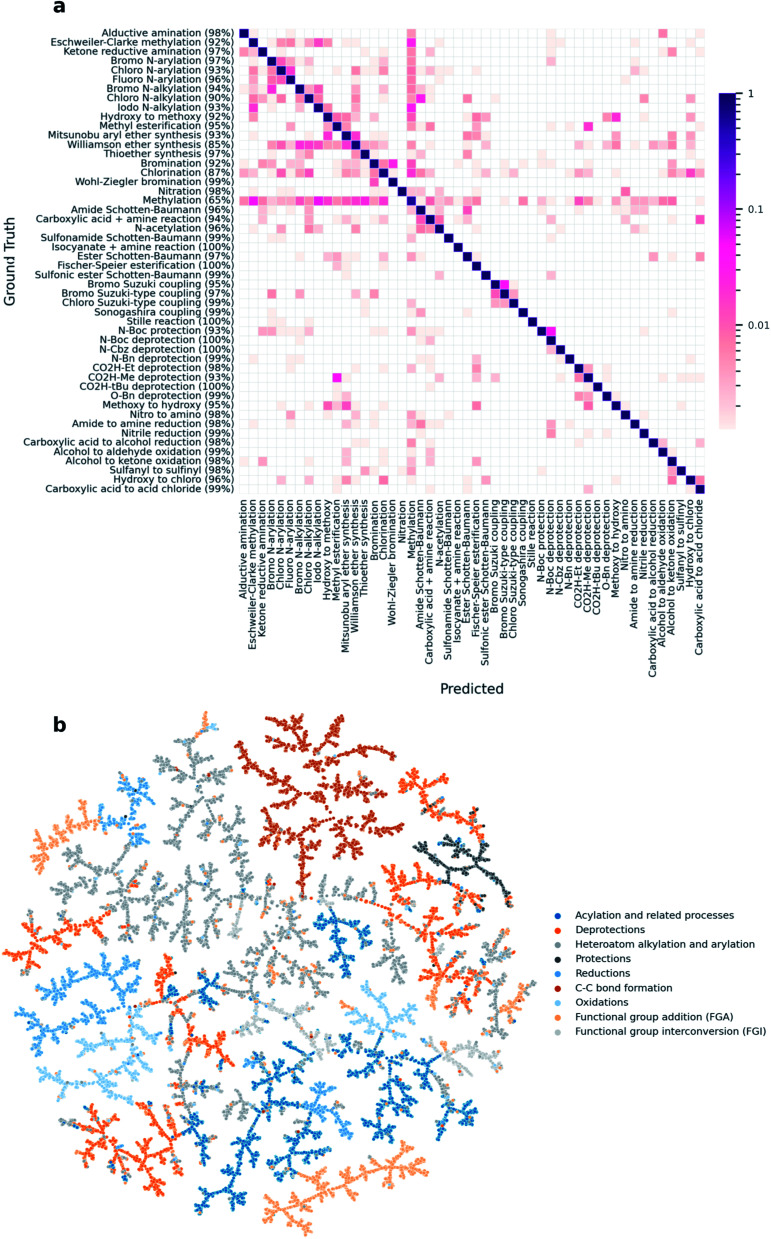
(a) An analysis of the per-class classification accuracy of an MLP model trained on *DRFP* shows that the high accuracy holds across most reaction classes (correctly predicted percentage per class in parentheses). (b) A TMAP created from *DRFP* fingerprints shows reactions being clustered by their respective super-class.

Inspired by the rxnfp-based reaction atlas from our previous work, we created a similar TMAP^25^ for the Schneider 50k data set using *DRFP*.^11,17^ The Schneider data set contains 50 000 reactions that are distributed evenly over 50 reaction classes, as annotated by the NameRxn tool.^16^ To analyse the performance of *DRFP* on the data set, we ran a classification task using the architecture and hyperparameters from the MLP used to classify the USPTO 1k TPL data set. After training on 10 000 reactions and evaluating on the remaining 40 000, the model reached an average classification accuracy of 0.956 (CEN = 0.053, MCC = 0.955). A confusion matrix across the 50 reaction classes in the data set shows that the high classification accuracy holds across the majority of the classes (Fig. 3a). Similar to the rxnfp-based classifier, our model reaches the lowest accuracy for the methylation reaction class, as methylation reactants often cause misclassifications.^11^ An example standing out in Fig. 3a are methylations involving iodomethane as a reagent with nitrogen-containing products being classified as iodo N-alkylations. Indeed, Schneider *et al.* also identified these classes as a source of misclassification and attributed them to the fact that some of the ground truth class assignments are ambiguous.^17^ The clustering of reactions by their super-classes in the TMAP (Fig. 3b) further shows that *DRFP* is well-suited for reaction classification tasks.

### 2.3 Reaction yield prediction

As a reaction regression task, we investigated yield prediction, where, given a chemical reaction, the percentage of the product that is formed compared to the theoretical maximum has to be predicted. One of the best studied yield data sets comes from a high-throughput experimentation study by Ahneman *et al.*,^6^ which contains the yields of 4608 palladium-catalysed Buchwald–Hartwig reactions with a fixed reactant and varying reagents. Numerous studies have previously used this data set model to evaluate the different machine learning models and representations (one-hot,^26^ physical,^6^ molecular^19^ and learned^10,11^ descriptors). The data set contains 10 random splits and 4 out-of-distribution test sets. In the out-of-distribution test sets, the split is made on the additives, which strongly influence the reactivity. Hence, the models have to extrapolate to unseen additives to perform well.

Comparing the yield prediction performance of *DRFP* to that of learned and physical descriptor-based fingerprints shows that this simple fingerprint is competitive, as it demonstrates consistent performance on all test sets. Averaging the 11 tests shown in Table 2, *DRFP* performs better than Yield-BERT, an augmented version of Yield-BERT, as well as a DFT-based method, in a yield prediction task on a data set of Buchwald Hartwig reactions. It also performs better than rxnfp in yield prediction of USPTO reaction data and a data set of Suzuki Miyaura reactions (Table 3).

**Table 2:** *R* ^2^ of yield prediction on Buchwald Hartwig reactions

*R* ^2^	DFT^6^	Yield-BERT^10^	Yield-BERT (aug.)^27^	*DRFP* (xgboost)
Rand 70/30	0.92	0.95 ± 0.005	**0.97 ± 0.003**	0.95 ± 0.005
Rand 50/50	0.9	0.92 ± 0.01	**0.95 ± 0.01**	0.93 ± 0.01
Rand 30/70	0.85	0.88 ± 0.01	**0.92 ± 0.01**	0.89 ± 0.01
Rand 20/80	0.81	0.86 ± 0.01	**0.89 ± 0.01**	0.87 ± 0.01
Rand 10/90	0.77	0.79 ± 0.02	**0.81 ± 0.02**	**0.81 ± 0.01**
Rand 5/95	0.68	0.61 ± 0.04	**0.74 ± 0.03**	0.73 ± 0.02
Rand 2.5/97.5	0.59	0.45 ± 0.05	0.61 ± 0.04	**0.62 ± 0.04**
Test 1	0.8	**0.84 ± 0.01**	0.8 ± 0.01	0.81 ± 0.01
Test 2	0.77	0.84 ± 0.03	**0.88 ± 0.02**	0.83 ± 0.003
Test 3	0.64	**0.75 ± 0.04**	0.56 ± 0.08	0.71 ± 0.001
Test 4	**0.54**	0.49 ± 0.05	0.43 ± 0.04	0.49 ± 0.004
Avg. 1–4	0.69	**0.73**	0.58 ± 0.33	0.71 ± 0.16
Avg. overall	0.75 ± 0.12	0.76 ± 0.17	0.778 ± 0.18	**0.786 ± 0.14**

**Table 3:** *R* ^2^ of yield prediction on Suzuki Miyaura reactions and on the USPTO data set that has been divided into gram scale and sub-gram scale yield subsets

*R* ^2^	Yield-BERT	*DRFP* (gradient boost)
Suzuki Miyaura	0.81 (±0.01)	**0.85** (±0.01)
USPTO (gram scale)	0.117	**0.13**
USPTO (sub-gram scale)	0.195	**0.197**

**Fig. 4:**
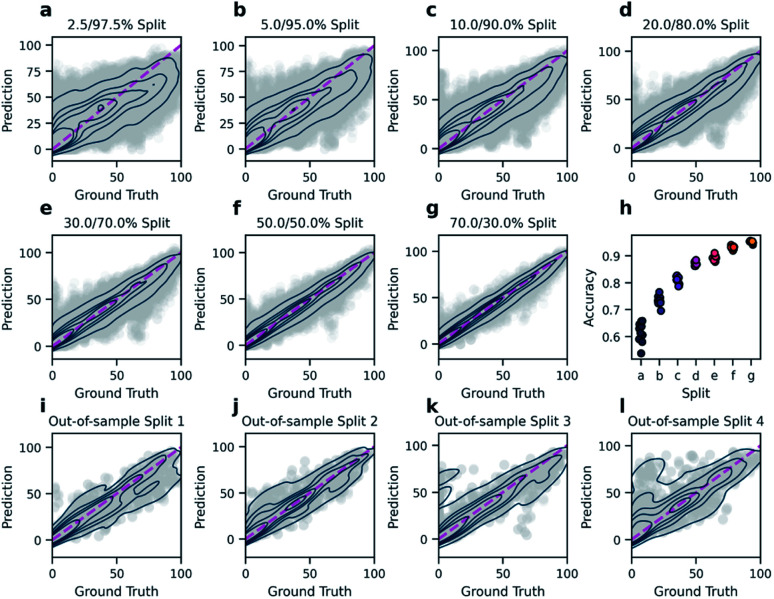
Regression plots for both the random split (a–g) and the out-of-sample (i–l) experiments. With few training data, the model generally predicts yields that are too high for low-yield reactions and yields that are too low for high-yield reactions (a–c). Further increase in the size of the training data yields diminishing increases in accuracy while the variance of accuracy between test sets decreases (d–h). In the out-of-sample-tests where the splits are defined by the isoxazole additives, performance varies heavily (i–l).

In order to predict reaction yields using *DRFP*, gradient boosting with early stopping was chosen as a machine learning technique. 10% of each training split was set aside and used to evaluate for early stopping. Hyperparameter optimisation was performed on five random splits (70/30). The resulting performance (*R*^2^) is then compared to the density functional theory (DFT) based fingerprint with a random forest regressor by Ahneman *et al.*,^6^ Yield-BERT, an extension of the learned rxnfp fingerprint with a regression layer, and an augmented variant of the latter (Table 2). The data set used is a collection of 3955 Pd-catalysed Buchwald–Hartwig C–N cross-coupling reactions from a high throughput experiment by Ahneman *et al.*^6^ For this data set, 11 splits were defined; seven splits where the relative size of the training set was decreased from 70 to 2.5% and four out-of-sample splits based on isoxazole additives. *DRFP* performs better on the random splits than the DFT-based fingerprint with random forests and Yield-BERT but is outperformed by the augmented Yield-BERT by a narrow margin. In the out-of-sample splits, *DRFP* performs better than the augmented version of Yield-BERT and the DFT-based method, yet the non-augmented Yield-BERT performs slightly better. When averaging over all 11 tests, *DRFP* performs best. Fig. 4 shows the regression plots for both the random split (a–g) and the out-of-sample (i–l) experiments. Under a low data regime, the xgboost model trained on *DRFP* tends to overestimate low-yield reactions and underestimate high-yield reactions (Fig. 4a–c), while the augmented Yield-BERT model generally predicts yields that are too low for low-yield reactions and too high for high-yield reactions.^27^ A similar tendency can be seen for the out-of-sample splits (Fig. 4a–c).

The performance of *DRFP* was further tested on a data set containing the yields of Suzuki–Miyaura reactions from a high-throughput experiment, and on reactions with associated yields from the USPTO reaction data set. The USPTO yield data set was split into a sub-gram and gram set to account for the different distributions of yields in the two subsets, as described by Schwaller *et al.*^10^ On both data sets, *DRFP* performed better than Yield-BERT (Table 3). Similar to the Buchwald–Hartwig reaction data, the difference between the two approaches is relatively small. In the case of the USPTO data set, both methods perform better on reactions with a sub-gram scale yield. A current limitation of *DRFP* is that it fails to distinguish between a reaction and its reverse, *e.g.* A + B → C + D and C + D → A + B. However, as the direction of the reaction is usually implied by the presence and absence of reactants, we consider this to be an edge-case that, if necessary, could be addressed in a specialised variant of the fingerprint.

Overall, *DRFP* reaches a compelling performance in yield prediction using a gradient boosting regressor that does not require hyperparameter tuning between different sets.

## 3 Conclusion

We have introduced a reaction fingerprint encoding scheme, *DRFP*, based on a simple 4-step process comprised of extracting circular *n*-grams, XORing, hashing, and folding. *DRFP* is capable of reaching state-of-the-art performance without extending the use of machine learning models from classification or regression tasks to the fingerprint creation task. Our results show that SMILES-encoded molecular graphs contain information that is sufficient for yield prediction by simple machine learning models, and that the explicit calculation of physics-based descriptors from the molecular graph as carried out by Ahnemann *et al.* is not necessary for this prediction task. While our method only slightly improves on the classification and prediction accuracies of other state-of-the-art methods, its value lies within its conceptual simplicity, low use of computational resources, and reproducibility. The fingerprint creation algorithm is available as a pypi package (drfp). Source code and documentation are available on GitHub (https://github.com/reymond-group/drfp).

## 4 Methods

### 4.1 Computational resources

We ran all of the training runs as well as the evaluations of the models on a DELL XPS Laptop with 16 GB of main memory, no dedicated GPU, and an 11th Gen Intel(R) Core(TM) i7-1165G7 @ 2.80 GHz CPU.

### 4.2 Molecular *n*-grams

Molecular *n*-grams are generated from SMILES, text-based encodings of the molecular graph, using the RDKit library. Given a radius *r*, we iterate over the heavy atoms in an input molecule and extract substructures centred on each atom with radii 0 to *r*, where a radius of 0 is the single central atom. These extracted substructures are then encoded as SMILES. A visual representation of this process for one atom in a molecule and *r* = 3 is shown in Fig. 1c. We denote the SMILES encodings of extracted substructures *molecular n-grams* in reference to *n*-grams found in bioinformatics.^28^ In addition, rings from the SSSR (smallest set of smallest rings) are extracted as *n*-grams as well. Compared to the atom pair-based approach by Schneider *et al.*,^17^ the *n*-grams-based fingerprint also captures stereochemistry, which can be defined using the SMILES notation. The pseudo-code for this process is shown in Box 1.

**Box 1: Generating molecular *n*-grams**

1:  *shingling* ← empty set

2:  **for***atom* in *molecule***do**

3:   **for***radius* = 0, …, *r***do**

4:    Add substructure with *radius* rooted at *atom* to *shingling* as SMILES

5:   **end for**

6:  **end for**

7:  **for***ring* in *sssr* (*molecule*) **do**

8:   Add substructure of *ring* to *shingling* as SMILES

9:  **end for**

### 4.3 Gradient boosting

For regression by gradient boosting, we used the Python library xgboost. Hyperparameter tuning was carried out on the rand 70/30 set of the Buchwald–Hartwig reaction data set. We applied the same hyperparameter values (n_estimators = 999 999, learning_rate = 0.01, max_depth = 15, min_child_weight = 8, colsample_bytree = 0.2125, subsample = 1) in all uses of xgboost. For each test, 10% of the training data were randomly selected as the validation set an removed from the training set. The validation data sets were used as evaluation sets for early stopping (20 rounds for all data sets with the exception of the USPTO, data for which 10 rounds were used to speed up the calculation).

### 4.4 *k*-Nearest neighbours classifier

The *k*-nearest neighbour classifier was implemented according to Schwaller *et al.*^11^ using faiss with *k* = 5.

### 4.5 Multilayer perceptron classifier

In addition to *DRFP* + 5-NN classifier, *DRFP* + multilayer perceptron (MLP) classifier was applied to the USPTO 1k TPL data set. The MLP was implemented using Tensorflow 2.4.1 and consists of an input layer the size of the input vector (2,048), a dense hidden layer of size 1664 and a tanh activation function, and a dense output layer with a softmax activation function. The loss function was sparse categorical cross-entropy. Adam was used as an optimiser. The model was trained over 10 epochs with a batch size of 64 on a CPU.

For the evaluation of AP3 256, the number of units in the hidden layer was changed to 1024, and the model was trained for 100 epochs.

### 4.6 Visualisations

All plots were created using the Python library matplotlib,^29^ the confusion matrix for the Schneider 50k classification task (Fig. 3a) was generated using the Python package pycm,^30^ and the TMAP coordinates for the Schneider 50k classification task (Fig. 3b) was created using the TMAP Python library.^25^

## Data availability

The source code, data and processing scripts for this paper, including the scripts to generate the fingerptins and the models are available at https://github.com/reymond-group/drfp. An release associated with the manuscript has been uploaded to Zenodo under the record https://zenodo.org/record/5268144#.YSeDXFuxWAk with DOI: 10.5281/zenodo.5268144.

## Author contributions

Daniel Probst: writing – original draft, visualization, software, formal analysis, investigation, methodology, validation Philippe Schwaller: writing – original draft, data curation, validation Jean-Louis Reymond: writing – review and editing, supervision, funding acquisition, project administration, resources.

## Conflicts of interest

There are no conflicts of interest to declare.

## Supplementary Material
